# Validation of the EQ-5D-5L and psychosocial bolt-ons in a large cohort of people living with multiple sclerosis in Australia

**DOI:** 10.1007/s11136-022-03214-y

**Published:** 2022-08-29

**Authors:** Julie A. Campbell, Hasnat Ahmad, Gang Chen, Ingrid van der Mei, Bruce V. Taylor, Suzi Claflin, Glen J. Henson, Steve Simpson-Yap, Laura L. Laslett, Kirsty Hawkes, Carol Hurst, Hilary Waugh, Andrew J. Palmer

**Affiliations:** 1 University of Tasmania, Menzies Institute for Medical Research, Hobart, TAS Australia; 2 Centre for Health Economics, Monash University, VIC, Australia; 3 School of Population and Global Health, Neuroepidemioloy Unit, The University of Melbourne, Melbourne VIC, Australia; 4 School of Population and Global Health, Health Economics Unit, The University of Melbourne, Melbourne VIC, Australia

**Keywords:** Multiple sclerosis, Australian MS Longitudinal Study, Multi-attribute utility instrument, EQ-5D-5L, AQoL-8D, EQ-5D-5L-Psychosocial, Cost-utility analysis, Sleep

## Abstract

**Background:**

Multiple sclerosis (MS) is an inflammatory, neurodegenerative disease of the central nervous system which results in disability over time and reduced quality of life. To increase the sensitivity of the EQ-5D-5L for psychosocial health, four bolt-on items from the AQoL-8D were used to create the nine-item EQ-5D-5L-Psychosocial. We aimed to externally validate the EQ-5D-5L-Psychosocial in a large cohort of people with MS (pwMS) and explore the discriminatory power of the new instrument with EQ-5D-5L/AQoL-8D.

**Methods:**

A large representative sample from the Australian MS Longitudinal Study completed the AQoL-8D and EQ-5D-5L (including EQ VAS) and both instruments health state utilities (HSUs) were scored using Australian tariffs. Sociodemographic/clinical data were also collected. External validity of EQ-5D-5L-Psychosocial scoring algorithm was assessed with mean absolute errors (MAE) and Spearman’s correlation coefficient. Discriminatory sensitivity was assessed with an examination of ceiling/floor effects, and disability severity classifications.

**Results:**

Among 1683 participants (mean age: 58.6 years; 80% female), over half (55%) had moderate or severe disability. MAE (0.063) and the distribution of the prediction error were similar to the original development study. Mean (± standard deviation) HSUs were EQ-5D-5L: 0.58 ± 0.32, EQ-5D-5L-Psychosocial 0.62 ± 0.29, and AQoL-8D: 0.63 ± 0.20. *N* = 157 (10%) scored perfect health (i.e. HSU = 1.0) on the EQ-5D-5L, but reported a mean HSU of 0.90 on the alternative instruments. The Sleep bolt-on dimension was particularly important for pwMS.

**Conclusions:**

The EQ-5D-5L-Psychosocial is more sensitive than the EQ-5D-5L in pwMS whose HSUs approach those reflecting full health. When respondent burden is taken into account, the EQ-5D-5L-Psychosocial is preferential to the AQoL-8D. We suggest a larger confirmatory study comparing all prevalent multi-attribute utility instruments for pwMS.

**Supplementary Information:**

The online version contains supplementary material available at 10.1007/s11136-022-03214-y.

## Introduction

### Multiple sclerosis

Multiple sclerosis (MS) is an inflammatory and neurodegenerative disease of the central nervous system (brain, optic nerves, and spinal cord) leading to increasing disability over time and reduced health-related quality of life (HRQoL) [1]. The *Atlas of MS* estimated that from 2013 to 2020, the global prevalence of MS increased by 500,000 to 2.8 million people [2]. In Australia, MS prevalence increased by 20% over 2010–2017 to 25,607 people [3]. MS generally presents in younger people between the ages of 20 and 40, when they are starting families and building careers [4]. The inflammatory demyelination of the brain and spinal cord causes lesions that manifest in a diverse array of symptoms including visual, sensory, cognitive, and sexual dysfunction, motor dysfunction and weakness, bowel or bladder continence issues, fatigue, anxiety, and depression [1]. Symptoms can appear individually or in concert and can result in marked declines in both physical and psychosocial health-related quality of life (HRQoL) [1, 5].

MS is associated with different clinical course phenotypes that include relapse-onset MS (ROMS), which results in a cycle of acute neurological impairment followed by complete or partial remission, and progressive-onset MS (POMS), which manifests as progressive neurological impairment without a relapse or remission [6]. Important differences are present between the broad classifications of progressing and relapsing. For example, the female-to-male patient ratio is nearer unity in POMS rather than a 1:4 ratio for ROMS, and POMS generally presents up to a decade later than ROMS. [7].

### Multi-attribute utility instruments to assess health state utilities as a measure of HRQoL

Healthcare resourcing decisions can be based on cost-utility analysis (CUA; a form of full health economic evaluation) for Health Technology Assessments (HTA) [8]. Several multi-attribute utility instruments (MAUIs) are available from which health state utilities (HSUs) can be derived as an input metric to CUA [8]. HSUs are used to reflect HRQoL and are values that measure the strength of preference for a particular health state, represented as a number between 0 and 1 where ‘0’ is anchored to death (or health states equivalent to being dead) and ‘1’ corresponds to perfect health. Health states worse than death are also possible, represented by negative utility values [9].

As well as being an input metric for CUA for resource allocation decisions [8], HSUs have also been shown to be independent predictors of patient outcomes, including all-cause mortality and development of complications [10]. Moreover, clinicians have found that measuring HSUs is of benefit to patients regarding clinical assessment, relationships, communication, and management [11].

Among MAUIs, the EQ-5D suite of instruments is the most widely used patient-reported questionnaire internationally including in HTAs [12]. The EQ-5D suite of instruments is used in over 63% of economic evaluations and recommended for CUA in over 85% of HTA guidelines worldwide [12]. However, due to its limited domains of psychosocial health (one domain of anxiety/depression), the EQ-5D-5L has been found to be deficient in capturing and assessing psychosocial health for people with complex and chronic diseases [13, 14]. Conversely, the less commonly used AQoL-8D is one of the most comprehensive MAUIs and is underpinned by 35 questions (25 of which relate to domains of psychosocial health). The AQoL-8D has been found to be preferentially sensitive to psychosocial health for people with complex and chronic disease, including for people with MS (pwMS) [13]. A recent systematic review that investigated the psychometric properties of MAUIs for pwMS found that in terms of discriminative ability, the EQ-5D-5L was not able to differentiate between those who were mildly or moderately disabled. However, the study also found that the AQoL-8D demonstrated good discriminative ability as it was able to differentiate between all levels of disability [5].

Our group has established that the AQoL-8D’s classification system works well for the complex symptomatology of MS [4, 15]. However, the 35 items of the AQoL-8D may be burdensome in some studies where multiple tests and surveys are required, such as randomised controlled trials. A recent study proposed including four response items as psychosocial bolt-on questions to extend the descriptive system of the EQ-5D-5L (hereafter, ‘EQ-5D-5L-Psychosocial’) to capture important elements of psychosocial health including vitality, relationships, sleep, and community connectedness [14]. This novel solution has two potential benefits compared to using the EQ-5D-5L or AQoL-8D alone. First, it allows for comparison purposes by using only responses to EQ-5D-5L items; meanwhile, it potentially would be more sensitive to diseases such as MS that have psychosocial burdens by using all nine items. Second, it substantially reduces the response burden as compared to AQoL-8D (i.e. respondents only answered nine items instead of 35 items). However, the new instrument has not been used in a large cohort of people with complex and chronic disease, such as MS, nor has it been compared to the source instruments of the EQ-5D-5L and AQoL-8D in the same cohort at the same time.

### Aims of this study

Against the backdrop of the development of the novel EQ-5D-5L-Psychosocial that has not been externally validated, nor used in a large study population with complex and chronic disease, this study had two aims. First, to externally validate the mapping function that is used to score the EQ-5D-5L-Psychosocial as outlined in the original development paper [14]. Second, we explored the discriminatory sensitivity of the EQ-5D-5L-Psychosocial compared to its source instruments (EQ-5D-5L and AQoL-8D) in a large, representative cohort of pwMS in Australia, the Australian Multiple Sclerosis Longitudinal Study (AMSLS) [3, 16].

## Methods

### Data sources: Australian MS Longitudinal Study (AMSLS)

The AMSLS is a large representative cohort of Australians with MS [17] comprising over 2600 active participants with self-reported MS. With the assistance of MS Research Australia and all Australian State and Territory MS Societies, recruitment to the AMSLS is ongoing to counter attrition [18].

#### Quality of life survey 2020

We conducted an extensive quality of life survey between 31 July and 30 September 2020 (2020 Quality of Life Survey) in the AMSLS cohort. The study was approved by the University of Tasmania’s Human Research Ethics Committee (number H0014183). All AMSLS participants provided informed consent. *N* = 2513 surveys were sent to active AMSLS participants (1875 online surveys, 613 paper-based surveys, and 25 phone surveys).

The order of individual MAUI questionnaires contained in the broader 2020 Quality of Life Survey was randomised and included the EQ-5D-5L [19] and AQoL-8D [20].

Other clinical and sociodemographic questions contained in the survey included age; sex; MS phenotype (relapsing–remitting MS [RRMS], secondary progressive MS [SPMS]) where RRMS and SPMS are combined as ROMS; and primary progressive MS (PPMS) and progressive-relapsing MS [PRMS] are combined as POMS); relapse in the past 12 months (number of relapses and current relapse); and disability severity measured by the Patient Determined Disease Steps (PDDS) [21].

#### Data from other AMSLS surveys

Special surveys are also disseminated to AMSLS participants every year [3, 22]. Other relevant sociodemographic information (namely education level and the number of years since MS diagnosis) was extracted from the AMSLS’ annual Disease Course Survey that was performed soon after the 2020 Quality of Life Survey.

To confirm the representativeness of our study sample, we compared the characteristics of respondents with non-respondents, and the broader AMSLS cohort.

### Multi-attribute utility instruments

#### EQ-5D-5L, EQ-5D-5L-Psychosocial, and AQoL-8D

Supplementary Table 1 and Supplementary Fig. 1A/1B outline the descriptive systems of the EQ-5D-5L, EQ-5D-5L-Psychosocial, and AQoL-8D, including the conceptual mapping for the EQ-5D-5L-Psychosocial. Table 1 also highlights the algorithmic ranges, number of health states, minimal important differences (MID) [13, 23], and population norms [24, 25] for the instruments (summary statistics described in Sect. ‘Respondent characteristics’).

**Table 1:** Characteristics and summary statistics for the EQ-5D-5L, EQ-5D-5L-Psychosocial, and AQoL-8D’s health state utilities and the EQ VAS scores

Instrument	EQ-5D-5L	EQ-5D-5L-Psychosocial	AQoL-8D	EQ VAS
*N* = 1683	0 = death 1.0 = perfect health	0 = death 1.0 = perfect health	0 = death 1.0 = perfect health	0 = worst health state 100 = best health state
Number of participants	1651	1635	1630	1618
Mean (SD)	0.58 (0.32)	0.62 (0.20)	0.63 (0.20)	68.67 (21.32)
Median (IQR)	0.63 (0.42–0.81)	0.63 (0.47–0.77)	0.63 (0.46–0.81)	75.00 (56.00–85.00)
Kurtosis*	3.5	2.4	2.0	3.0
Observed range	−0.68–1.0	0.05–1.0	0.10–1.0	0–100
Algorithmic range** (Australian value sets)	−0.68–1.0	0.046–1.0	0.09–1.0	NA
Potential health states	3125	1,953,125	2.4*10^23^	NA
Population norms for the general Australian population	0.91 (0.14)	NA	0.80 (0.19)	78.55 (16.57)
Minimal important difference (MID)	0.04	NA	0.08	8.61–10.82
Participants on ceiling (*n*, %)	157 (10%)	7 (0.004%)	7 (0.004%)	0
Participants on floor (*n*, %)***	3 (0.002%)	1 (0.001%)	0	0

*Kurtosis < 3 Platykurtic; > 3 Leptokurtic; = 3 Mesokurtic

**EQ-5D-5L Australian value set Norman et al. 2013; EQ-5D-5L-Psychosocial Chen et al. 2020, AQoL-8D Richardson et al. 2014

***Participants on the floor calculated for a health state utility ≤ −0.06 for the EQ-5D-5L, < 0.05 for the AQoL-8D and EQ-5D-5lpsychosocial

*NA*: not applicable

The EQ‐5D-5L asks participants to indicate whether they have problems on a five-level scale for each of the five dimensions of health: mobility, self‐care, usual activities, pain/discomfort, and anxiety/depression. The EQ-5D-5L describes 3125 health states and was developed to address the limited sensitivity (lack of descriptive richness and ceiling effects) of the EQ-5D-3L [19] which describes 243 health states. The algorithmic range for most of the instrument’s country-specific value sets describes HSUs ranging from < 0 to 1.0 [9]. The EQ-5D-5L also uses a visual analogue scale (EQ VAS) in which participants rate their current health state on a scale of 0 to 100 (worst to best imaginable health) [13] (Supplementary Table 1, Table 1).

The AQoL-8D was originally developed to achieve sensitivity not only in health states affected by physical disorders, but also in those affected by mental disorders [26]. The AQoL-8D instrument contains 35 items in eight dimensions and was derived using psychometric methods for achieving content validity. Three of the dimensions (independent living, pain, senses) load to a physical super-dimension; the other five (mental health, happiness, coping, relationships, and self-worth) load to a mental super-dimension. The size of the instrument means that it can define billions of health states [26] (Supplementary Fig. 1A, Table 1).

The EQ-5D-5L-Psychosocial was developed to address the psychosocial gaps in the EQ-5D-5L by including the additional dimensions of vitality, relationships, sleep, and social isolation, which were adopted from four bolt-on questions from the AQoL-8D (Supplementary Table 1, Supplementary Fig. 1A/1B, Table 1) [14]. The developmental phase of the new instrument found that vitality was the most important dimension with regard to HRQoL [14] (Supplementary Fig. 1B). Given the dominant position of the EQ-5D-5L in applied studies, the developers suggested that identifying a set of bolt-on dimensions that captured the psychosocial aspects of health would serve as a realistic alternative (at least in the short run) for developing a completely new extended generic preference-based measure [14]. The scoring algorithm was developed from a mapping analysis that mapped responses to nine items (five EQ-5D-5L and four bolt-on items) onto the AQoL-8D utilities. The developers also indicated that there was a need for external validation of the proposed scoring algorithm [14].

A minimal important difference (MID) is the smallest difference in score in the outcome of interest that patients perceive as beneficial and would mandate a change in the patient’s management [11, 27]. A composite MID measure for the EQ-5D-5L for chronic health conditions is 0.04 utility points [27]. A MID for the AQoL-4D is 0.06 utility points (95% confidence interval 0.03–0.08 utility points) [28]. For this study, and upon external validation of the new instrument, we assume that the MID for the new instrument will adopt a MID that aligns with the AQoL suite of instruments of 0.06 utility points [28].

### Statistical methods

The primary outcome measure of this study was HRQoL, captured and assessed by the HSUs generated using Australian tariffs. A secondary measure was the EQ VAS score. In summary, we first investigated the external validity of the novel EQ-5D-5L-Psychosocial’s HSUs for our AMSLS study population, compared with the study population and internal validation of the original development paper [14]. Second, we conducted an exploratory head-to-head comparison of the discriminatory sensitivity of the HSUs generated by the EQ-5D-5L-Psychosocial with its two source questionnaires from the EQ-5D-5L and AQoL-8D for our representative study population of pwMS.

#### External validation of the EQ-5D-5L-Psychosocial scoring algorithm

We investigated the external validity of EQ-5D-5L-Psychosocial’s scoring algorithm (developed based on a mapping analysis) using our AMSLS study population, as well as compared the goodness-of-fit statistics against the internal validation results reported in the original development paper [14]. The mean absolute error (MAE) was chosen as the key statistic for measuring the average prediction error [29]. It has been suggested that the MAE is the most natural and unambiguous measure of average error magnitude [29]. The MAE described in the development paper of the final mapping function was 0.058 [14]. We also reported the strength of Spearman’s correlation and agreements between the EQ-5D-5L-Psychosocial and AQoL-8D HSU. A Spearman’s rho of > 0.7 is considered strong and > 0.9 is considered very strong [30].

#### MAUI comparisons

##### Descriptive analyses

Summary data describing the sociodemographic characteristics of the participants are presented as means with standard deviations (SD) for continuous variables and as percentages with frequency counts for categorical variables.

Questionnaire completion was assessed with the individual responses to MAUI items (questions) using counts and proportions.

Summary statistics were generated for HSUs and EQ VAS scores for the overall sample and then stratified by sociodemographic characteristics including age (< 35 years, 35–44, 45–54, 55–64, and > 65); sex; Australian state or territory of usual residence, educational attainment (primary, secondary, occupation certificate, university (bachelors), university (postgraduate)); MS phenotype (progressive and relapsing classifications) and disability severity (no disability, mild, moderate, and severe; see further detail below), and years since diagnosis of MS (< 10 years, 10–14 years, 15–19 years, 20–29 years, > 30 years) to broadly reflect expected disability severity classifications since the time of diagnosis and to also provide some equivalency of groupings for further investigation of unadjusted HSUs.

We also investigated the frequency distribution of the individual HSUs for each instrument including associated kurtosis.

##### Ceiling effects/floor effects

In regard to ceiling effects, we examined the counts and proportions for people who scored perfect health (HSU = 1.0) for the EQ-5D-5L and AQoL-8D and compared the individual HSUs and summary statistics of the HSUs generated for these participants on the EQ-5D-5L-Psychosocial, as described in our previously published work [13]. We also investigated participant’s responses to the individual items of the alternate instruments [13].

We adopted the same methodology for the examination of floor effects. In assuming the floor effect, we note that the algorithmic range for the Australian tariff of the EQ-5D-5L is substantially broader than the alternate instruments, being almost 0.5 utility points larger and scoring in the negative range namely −0.676 to 1.0 compared to EQ-5D-5L-Psychosocial (0.046–1.0) and AQoL-8D (0.09–1.0) (Table 2). Therefore, we assumed the floor effect to be < -0.05 utility points for the EQ-5D-5L and < 0.1 utility points for the AQol-8D.

**Table 2:** Clinical and sociodemographic characteristics of respondents versus non-respondents

*N* = 2513	Respondents	Non-respondents
Characteristics	(*N* = 1683)	(*N* = 830)
*Age at the time of survey*		
Average in years (*n*)	58.6 (1683)	55.6 (830)
*Sex % (n)*		
Male	20.4 (343)	21.8 (181)
Female	79.6 (1340)	78.2 (649)
*Age group % (n)*		
< 35	1.8 (31)	4.7 (39)
35–44	9.3 (157)	15.5 (129)
45–54	24.7 (415)	23.5 (195)
55–64	32.1 (540)	30.8 (256)
65 +	32.1 (540)	25.4 (211)
*State/Territory of usual residence % (n)*		
New South Wales	28.1 (473)	32.2 (267)
Victoria	29.3 (493)	23.6 (196)
Queensland	12.4 (209)	18.6 (154)
South Australia	9.9 (167)	8.4 (70)
Western Australia	9.8 (165)	9.4 (78)
Australian Capital Territory	4.0 (67)	3.0 (25)
Tasmania	5.7 (96)	4.2 (35)
Northern Territory	< 1.0 (1)	< 1.0 (3)
*Education Levels % (n)*		
Primary	< 1.0 (8)	< 1.0 (7)
Secondary	24.6 (400)	26.1 (203)
Occupation Certificate	33.5 (545)	30.3 (236)
University (bachelors)	21.0 (341)	22.3 (174)
University (Postgrad)	16.7 (272)	16.9 (132)
Other	3.7 (60)	3.5 (27)
MS type % (n)*		
PPMS	11.3 (190)	NA
RRMS	62.8 (1056)	NA
SPMS	14.2 (239)	NA
PRMS	2.5 (42)	NA
Unsure	8.0 (134)	NA
*Disability severity % (n)*		
No disability	23.9 (402)	NA
Mild disability	20.4 (343)	NA
Moderate disability	36.1 (608)	NA
Severe disability	18.8 (317)	NA
*MS duration since diagnosis % (n)*		
Average in years (*n*)	19.0 (1393)	16.9 (683)

*Multiple sclerosis (MS) phenotype = PPMS, primary progressive MS; RRMS, relapsing–remitting MS; SPMS, secondary progressive MS; PRMS, progressive-relapsing MS

*NA* not available in the 2020 Quality of Life survey

##### Bland Altman analysis

To determine the interchangeability between the instruments, pairwise agreements between the HSUs for each instrument for each participant were assessed through the Bland–Altman method of differences [31]. In regard to the Bland–Altman plots, the difference between the two measures was plotted against the mean measurement for those two instruments for each individual along with the limits of agreement (the range of values that would be expected to include 95% of individual differences) [31].

##### Discriminatory sensitivity of MAUI HSUs: disability severity and MS type

Disability was assessed with the PDDS, which was then mapped to the gold-standard Expanded Disability Status Scale (EDSS) for four classifications of MS-related disability severity classified as no disability (EDSS level: 0), mild disability (EDSS > 0–3.5), moderate disability (EDSS > 3.5–6), and severe disability (EDSS > 6–9.5) [4, 21]. PDDS and EDSS both primarily assess mobility and physical health [32]. For the purposes of comparing HSUs of the three instruments for the participant sample, MS type was classified as progressive (POMS) and relapsing (ROMS).

We assessed the sensitivity of the instruments in detecting the differences of different disability severities (mild, moderate, and severe) using a regression analysis in which a set of confounding factors (age and sex) were controlled for. To facilitate cross-instrument comparisons, the standardised coefficients are reported.

All statistical analyses were conducted in STATA/SE 17.0 (StataCorp, College Station, USA) and R 4.0.2.

## Results

### Respondent characteristics

**Fig. 1:**
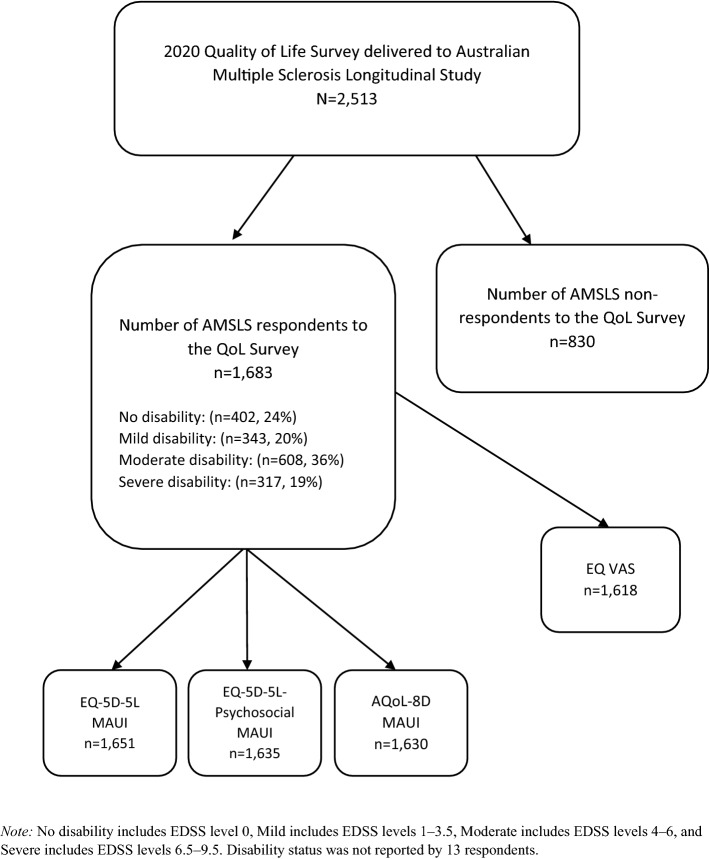
Flow of participants with multiple sclerosis into the study and the generation of health state utilities for the EQ-5D-5L, AQoL-8D, and EQ-5D-5L-Psychosocial multi-attribute utility instruments (MAUI) and EQ VAS scores

Figure 1 provides a summary of the flow of participants into the study, including the number of participants for whom we could generate a HSU for the two source instruments and the EQ-5D-5L-Psychosocial. Of the 2513 invitations sent to active participants of the AMSLS, 1683 pwMS responded to the survey (67%), this response rate slightly exceeding the response rates of other targeted AMSLS surveys [16]. HSUs could be generated for an average of 97% of participants: EQ-5D-5L (*n* = 1651), EQ-5D-5L-Psychosocial (*n* = 1635), and AQoL-8D (*n* = 1630). Supplementary Table 2 provides counts for missing responses for the EQ-5D-5L (*n* = 54 of *n* = 8415 possible responses to individual questions) and AQoL-8D (*n* = 314 of *n* = 58,905 possible responses to individual questions).

Table 2 provides the sociodemographic characteristics of respondents and non-respondents. Our sample was largely representative of the broader AMSLS cohort. Respondents were slightly older by 3 years (mean age 58.6 years). Ratio of males to females was similar with almost 80% female; typical for MS. Education levels were also similar with almost 70% of respondents holding an occupation certificate or tertiary degree. More specifically, participants were mainly female (79.6%), middle-aged (58.6 years), and educated (almost 75% obtaining an occupational diploma or tertiary degree). In regard to disability severity categories according to the EDSS classifications, 23.9% had no disability, 20.4% mild disability, 36.1% moderate disability, and 18.8% severe disability. *N* = 207 participants were reporting a current relapse event and 62.8% of the sample were people with RRMS (Table 2).

### External validation of the EQ-5D-5L-Psychosocial algorithm for pwMS

We first compared the MAE of the final mapping function from the development dataset of the new instrument of 0.058 and 0.059 (from the two internal validation tests of two samples of *n* = 1000 and *n* = 5000) with that our AMSLS study population which was 0.063. Figure 3 further compares the distribution of the prediction errors and scatterplots of the AQoL-8D and EQ-5D-5L-Psychosocial.

**Fig. 2:**
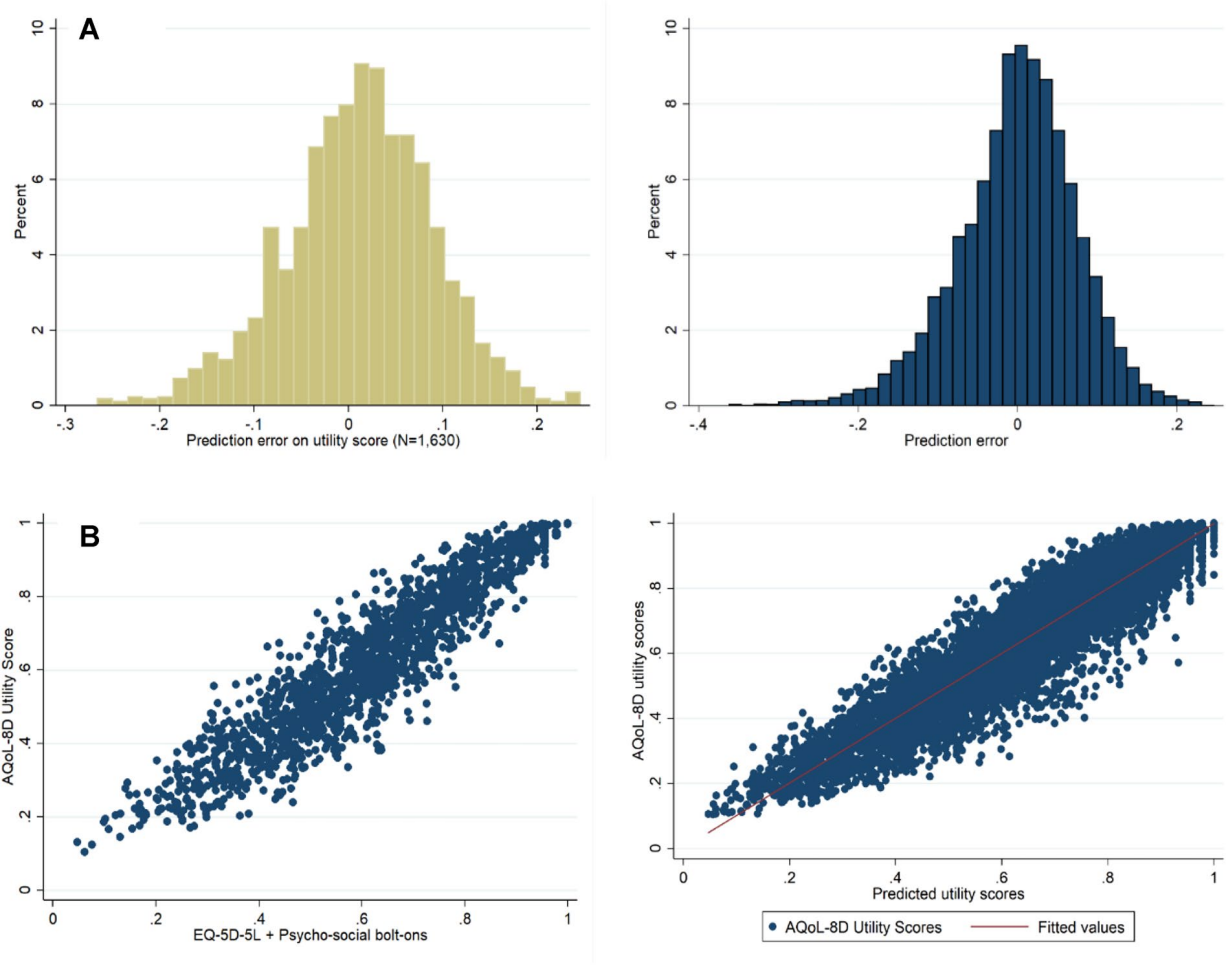
External validation of the EQ-5D-5L-Psychosocial with the Australian Multiple Sclerosis Longitudinal Study 2020 Quality of Life Survey (*n* = 1630). **A** Distribution of the prediction error of health state utilities from the AQoL-8D to the EQ-5D-5L-Psychosocial for external validation with the AMSLS compared to distribution of the prediction error from the original development of the instrument (Chen et al. 2020) and **B** Scatterplot between the EQ-5D-5L-Psychosocial and the AQoL-8D reflecting the performance of the original development paper

The distribution of the prediction error of health state utilities between the observed AQoL-8D and the predicted utilities from the EQ-5D-5L-Psychosocial for external validation with the AMSLS is also similar to that of the original development study (Fig. 2A). The scatterplot between the observed AQoL-8D HSU and the EQ-5D-5L-Psychosocial HSU revealed a very high correlation (*r *= 0.93, Table 3) and the performance was very similar to that of the original development study (Fig. 2B).

**Table 3:** Spearman’s correlation matrix of EQ VAS and the three multi-attribute utility instruments (EQ-5D-5L, AQoL-8D and EQ-5D-5L-Psychosocial)

	EQ VAS	EQ-5D-5L	AQoL-8D	EQ-5D-5L-Psychosocial
EQ VAS	1.00			
EQ-5D-5L	0.59	1.00		
AQoL-8D	0.64	0.76	1.00	
EQ-5D-5L-Psychosocial	0.66	0.82	0.93	1.00

All values were significant at *p* < 0.05

### Performance of using EQ-5D-5L-Psychosocial against EQ-5D-5L and AQoL-8D for pwMS

#### Summary statistics

Supplementary Fig. 2 shows the overall distributions of the instruments’ HSUs and the EQ VAS. In regard to the distribution of the individual utilities, the EQ-5D-5L was left skewed and had higher ceiling effects than the AQoL-8D and EQ-5D-5L-Psychosocial. Table 4 presents summary statistics for the HSUs for the overall cohort and stratified by sociodemographic and clinical characteristics. The EQ-5D-5L generated substantially lower mean HSUs compared to the AQoL-8D and EQ-5D-5L-Psychosocial for people with severe disability and for people who were experiencing an acute relapse of their MS symptoms. On the other hand, for people with no disability or mild disability, the EQ-5D-5L generated higher HSUs than the alternative instruments. In regard to ROMS (relapsing MS phenotype) versus POMS (progressive MS phenotypes), the EQ-5D-5L showed a similar trend with a substantially reduced EQ-5D-5L HSU compared to the alternative instruments for people with progressive forms of MS. Notably, higher age quintiles and people who had been diagnosed with MS at least 30 years ago also had this pattern (Table 4).

**Table 4:** Summary statistics for the EQ-5D-5L, EQ-5D-5L-Psychosocial and AQoL-8D health state utilities

*N* = 1683	EQ-5D-5L					EQ-5D-5L-Psychosocial					AQoL-8D				
	Mean	SD	Min	Max	N	Mean	SD	Min	Max	N	Mean	SD	Min	Max	N
Overall sample	0.58	0.32	−0.68	1.00	1651	0.62	0.20	0.05	1.00	1635	0.63	0.20	0.10	1.00	1630
*Sex*															
Female	0.59	0.32	−0.68	1.00	1321	0.62	0.20	0.05	1.00	1311	0.62	0.21	0.11	1.00	337
Male	0.51	0.33	−0.68	1.00	330	0.61	0.19	0.06	1.00	324	0.63	0.22	0.12	0	1332
*Age group (years)*															
< 35	0.74	0.18	0.34	1.00	31	0.71	0.16	0.39	1.00	31	0.73	0.17	0.37	1.00	31
35–44	0.67	0.27	−0.52	1.00	155	0.63	0.19	0.15	1.00	154	0.64	0.21	0.17	1.00	156
45–54	0.63	0.29	−0.35	1.00	410	0.62	0.20	0.10	1.00	410	0.63	0.22	0.17	1.00	414
55–64	0.57	0.32	−0.68	1.00	529	0.61	0.20	0.05	1.00	525	0.62	0.21	0.12	1.00	538
65 +	0.50	0.34	−0.68	1.00	526	0.61	0.19	0.06	0.98	515	0.62	0.21	0.11	1.00	530
*Education level*															
Secondary School and below	0.51	0.35	−0.68	1.00	418	0.59	0.21	0.06	0.98	414	0.59	0.22	0.11	1.00	424
Occupational certificate	0.56	0.32	−0.60	1.00	576	0.61	0.19	0.05	1.00	571	0.61	0.21	0.14	1.00	582
University bachelors	0.62	0.30	−0.68	1.00	357	0.64	0.20	0.13	1.00	354	0.66	0.21	0.21	1.00	360
University postgrad	0.65	0.28	−0.18	1.00	289	0.64	0.19	0.14	1.00	285	0	0	0	1.00	292
*Disability severity*															
No	0.85	0.16	−0.22	1.00	394	0.79	0.14	0.10	1.00	392	0.82	0.15	0.19	1.00	401
Mild	0.71	0.20	−0.20	1.00	342	0.66	0.16	0.24	0.98	341	0.67	0.17	0.23	1.00	343
Moderate	0.52	0.22	−0.27	1.00	596	0.53	0.17	0.11	0.96	588	0.54	0.18	0.18	1.00	602
Severe	0.18	0.31	−0.68	0.83	308	0.50	0.18	0.05	0.90	303	0.51	0.19	0.11	0.97	312
*MS phenotype*															
Progressive	0.36	0.34	−0.68	1.00	225	0.54	0.19	0.10	1.00	219	0.55	0.21	0.17	1.00	230
Relapsing	0.61	0.30	−0.68	1.00	1289	0.62	0.20	0.05	1.00	1280	0.64	0.21	0.11	1.00	1298
*DMT current*															
Yes	0.62	0.29	−0.57	1.00	846	0.63	0.20	0.08	1.00	842	0.62	0.29	−0.57	1.00	846
No	0.51	0.35	−0.68	1.00	477	0.60	0.20	0.06	1.00	469	0.61	0.21	0.11	1.00	483
*Current MS-related Relapse*															
Yes	0.43	0.32	−0.57	1.00	202	0.50	0.19	0.05	0.96	199	0.48	0.20	0.12	1.00	205
No	0.66	0.28	−0.35	1.00	1111	0.66	0.19	0.11	1.00	1104	0.68	0.20	0.18	1.00	1120
*Years since first diagnosed with MS*															
< 10 years	0.61	0.30	−0.35	1.00	229	0.60	0.20	0.14	1.00	225	0.60	0.22	0.18	1.00	232
10–14 years	0.63	0.30	−0.60	1.00	367	0.64	0.19	0.10	1.00	366	0.65	0.21	0.18	1.00	370
15–19 years	0.61	0.31	−0.68	1.00	419	0.63	0.20	0.06	0.98	417	0.64	0.21	0.11	1.00	421
20–29 years	0.55	0.34	−0.57	1.00	440	0.60	0.20	0.05	1.00	436	0.61	0.21	0.12	1.00	449
> 30 years	0.43	0.32	−0.34	1.00	181	0.59	0.18	0.13	0.96	176	0.60	0.20	0.15	0.99	182

#### Ceiling and floor effects

**Fig. 3:**
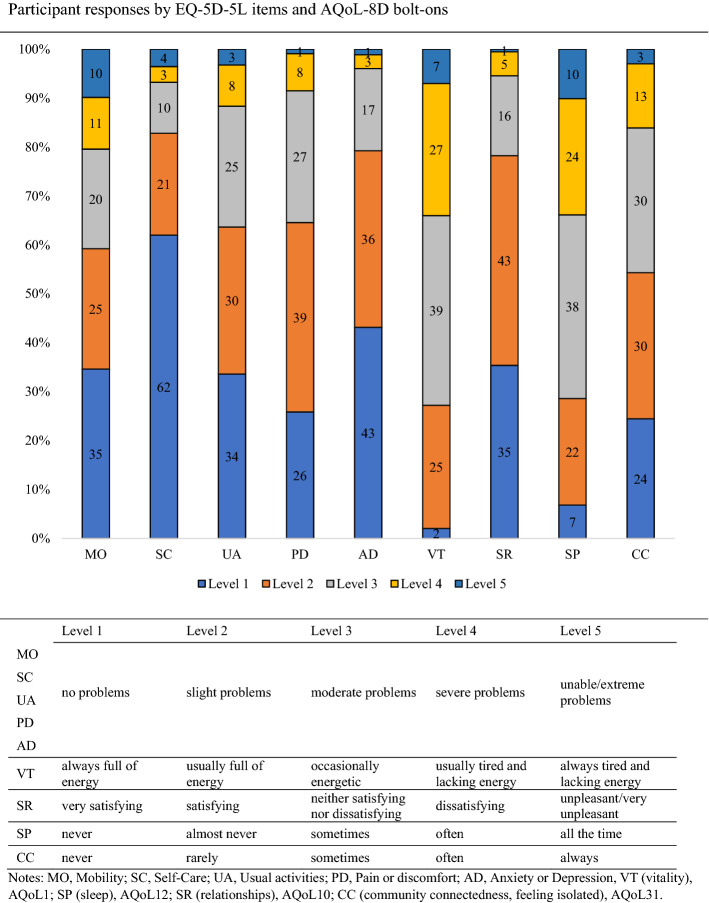
Participant’s responses to the individual items as proportions (%) for the EQ-5D-5L (*n* = 1651) (mobility, selfcare, usual activities, pain/discomfort, anxiety/depression) and AQoL-8D bolt-ons (*n* = 1635) (vitality, relationships, sleep, community connectedness)

Figure 3 provides the proportions of responses to the nine items of the EQ-5D-5L-Psychosocial. Responses to the bolt-on questions regarding vitality, social relationships, sleep, and community connectedness reveal that most pwMS responded to these questions at levels 2 and above (for maximum levels of 4–6). Sleep and vitality had the highest number of responses for levels 4 and 5 with over 30% of responses at these levels.

**Fig. 4:**
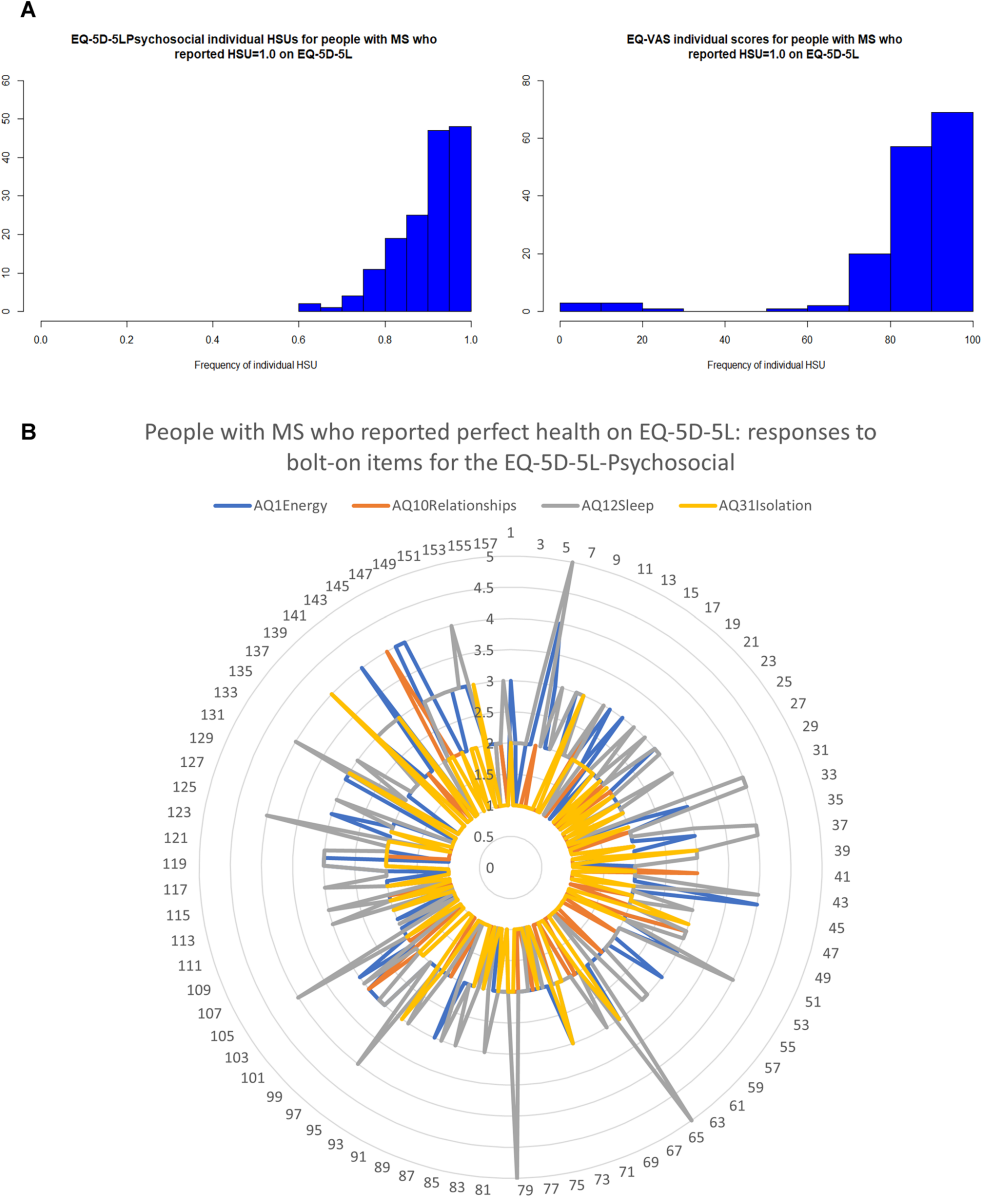
Investigation of ceiling effects for the EQ-5D-5L (*n* = 157). **A** EQ-5D-5L-Psychosocial individual health state utilities and EQ VAS scores for people with MS (*n* = 157) who reported a health state utility of 1.0 (perfect health) on the EQ-5D-5L. **B** People with MS (*n* = 157) who reported a health state utility of 1.0 (perfect health) individual responses to the AQ0L-8D bolt-on items of vitality (AQ1 energy), relationships (AQ10 relationships), sleep (AQ12 sleep), and social isolation (AQ31 isolation) where 1 = best response and 5 = worst response

Table 5 (Supported by Supplementary Table 3) describes the summary statistics and distributions across the disability severity classifications for people who scored perfect health on the EQ-5D-5L against the alternative instruments. Of the *n* = 1651 pwMS who generated a utility value for the EQ-5D-5L, *n* = 157 (10%) generated a HSU of 1.0 (perfect health). The distributions of the individual HSUs for these people with the EQ-5D-5L-Psychosocial are shown in Fig. 4 with Table 5 revealing a range of 0.620 to 1.0 and mean (SD) 0.90 (0.08). Notably, six of these participants reported a moderate disability. In contrast, for the EQ-5D-5L-Psychosocial, of the 1635 participants who generated a HSU, only *n* = 7 (0.004%) reported an HSU of 1.0. This result was also mirrored for the AQoL-8D for the 1630 participants who generated a HSU: only *n* = 7 (0.004%) reported an HSU of 1.0 (Table 1).

**Table 5:** Summary statistics for (*n* = 157) participants who reported full health (health utility = 1.0) on the EQ-5D-5L for the alternate instruments of the EQ-5D-5L-Psychosocial and AQoL-8D and the EQ VAS; and their EDSS disability severity classifications (supported by Supplementary Table 2)

Variable	EQ-5D-5L-Psychosocial	AQoL-8D	EQ VAS
Age (years)	55.4	55.4	55.4
Female (*n*, %)	136 (87%)	136 (87%)	136 (87%)
*HSU or EQ VAS score*			
Mean (SD)	0.90 (0.08)	0.91 (0.09)	85.60 (17.86)
Median (IQR)	0.92 (0.86–0.96)	0.94 (0.88–0.97)	90.0 (85.0–95.25)
Range	0.62–1.00	0.63–1.00	0–100
*Disability severity (n, %)*			
EDSS Normal	119 (76%)	119 (76%)	119 (76%)
EDSS Mild	31 (20%)	31 (20%)	31 (20%)
EDSS Moderate	6 (4%)	6 (4%)	6 (4%)
EDSS Severe	0	0	0

Expanded Disability Status Scale (EDSS) our classifications of MS-related disability severity classified as no disability (EDSS level: 0), mild disability (EDSS > 0–3.5), moderate disability (EDSS > 3.5–6), and severe disability (EDSS > 6–9.5)

Table 6 (supported by Fig. 4) highlights the participant’s responses to the individual items as proportions for AQoL-8D bolt-ons (vitality, relationships, sleep, community connectedness) for those people who scored perfect health on the EQ-5D-5L (*n* = 157). This analysis revealed that for pwMS who are regarded as full health according to the EQ-5D-5L classification system for the five EQ-5D-5L items, when asked questions that directly relate to psychosocial health, the proportions of responses in levels 2 to 5 are substantial. Most importantly, these participants rated sleep as crucial to their psychosocial health (despite reporting perfect health on the EQ-5D-5L), with almost 80% of these participants rating sleep quality as reduced from levels 2 to 5.

**Table 6:** Proportions of responses across the four AQoL-8D bolt-on items for the EQ-5D-5L-Psychosocial for participants who reported full health (health utility = 1.0) on the EQ-5D-5L

Bolt-on dimensions	Level of response				
*N* = 157	Best response				Worst response
*n* (%)	Level 1	Level 2	Level 3	Level 4	Level 5
Vitality	19 (12%)	105 (67%)	28 (18%)	5 (3%)	0
Relationships	105 (67%)	45 (29%)	5 (3%)	2 (1%)	0
Sleep	22 (14%)	74 (47%)	47 (30%)	11 (7%)	3 (2%)
Community*	100 (64%)	47 (30%)	9 (6%)	1 (1%)	0

^*^Community % adds to 101 due to rounding; Bolt-on dimensions: vitality (AQoL-8D Q1), relationships (AQoL-8D Q10), sleep (AQoL-8D Q12), community connectedness/isolation (AQoL-8D Q31)

In regard to floor effects, for the EQ-5D-5L HSUs for health states less than −0.06, only *n* = 3 participants scored on the floor of the algorithmic range. However, 17 participants scored a HSU less than zero for the EQ-5D-5L; this is not possible for the EQ-5D-5L-Psychosocial and AQoL-8D with possible ranges of 0.046–1.00 and 0.09–1.00, respectively. Therefore, the summary HSUs for participants with severe disability were substantially lower for the severe disability category (mean 0.18) than the AQoL-8D (mean 0.50) and EQ-5D-5L-Psychosocial (mean 0.50) HSUs for this category.

#### Interchangeability

**Fig. 5:**
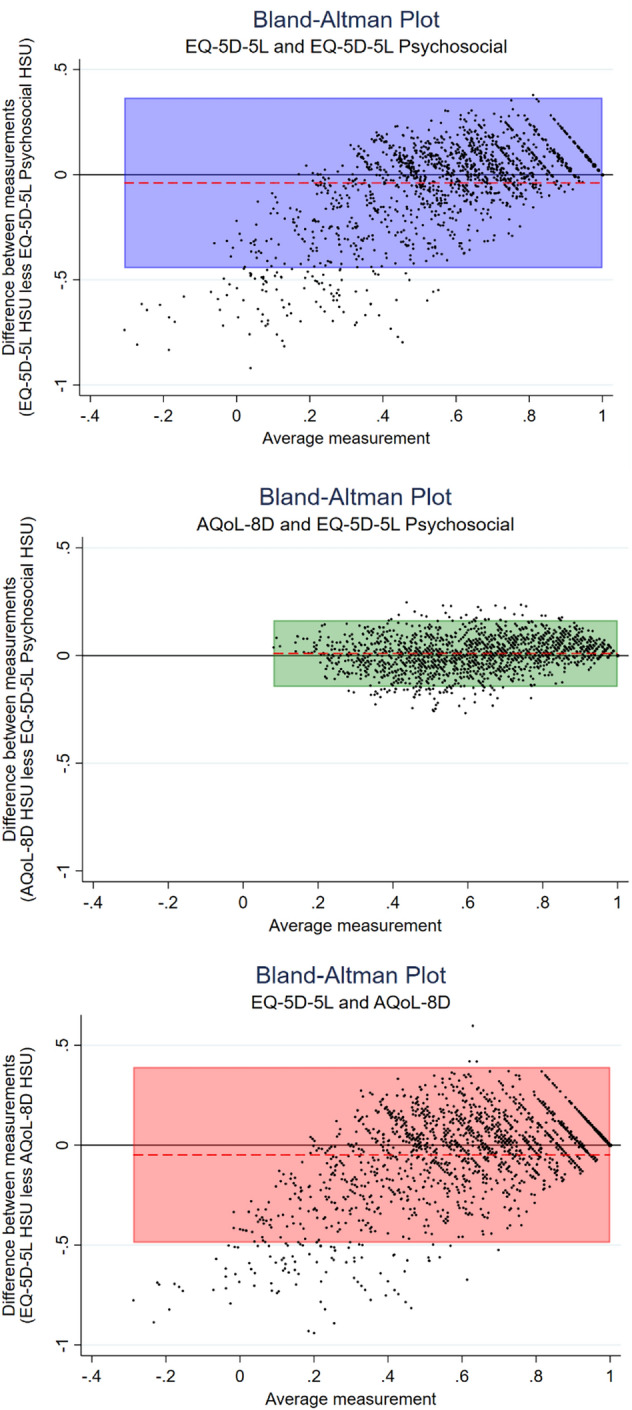
Investigation of the interchangeability of the EQ-5D-5L, EQ-5D-5L-Psychosocial, and AQoL-8D using Bland Altman analysis (highlighted section is the area of agreement)

Figure 5 shows Bland–Altman analysis regarding the pairwise agreement. In regard to the EQ-5D-5L and EQ-5D-5L-Psychosocial, the mean HSUs for these instruments for the overall sample had a difference that met the MID for the EQ-5D-5L of 0.04 utility points. Bland–Altman analysis of these two instruments also provided evidence that the two instruments are not interchangeable with a relatively wide level of agreement and systematic variation revealed in the Bland–Altman plot.

In regard to the EQ-5D-5L-Psychosocial and AQoL-8D, the mean HSU difference for these instruments for the overall sample did not meet the MID for the AQoL-8D. Additionally, the Bland–Altman analysis for the AQoL-8D and EQ-5D-5L-Psychosocial revealed no systematic variation and a relatively narrow limit of agreement suggesting that there was a pairwise agreement between the two instruments.

Overall our results demonstrate that the EQ-5D-5L and EQ-5D-5L-Psychosocial are not interchangeable; however, the AQoL-8D and EQ-5D-5L-Psychosocial are interchangeable.

#### Sensitivity

Table 7 (Panel A) shows the standardised coefficients of key variables of interest from the regression analyses. Based on the absolute values of the standardised coefficients, this analysis established that between the reference level of no disability and mild or moderate levels of disability, the EQ-5D-5L-Psychosocial and AQoL-8D had higher discriminatory sensitivity compared to the EQ-5D-5L. In regard to people with severe disability, the EQ-5D-5L is more sensitive.

**Table 7:** Comparisons of standardised estimates for participants who generated a health state utility for the EQ-5D-5L, EQ-5D-5L-Psychosocial (using the Australian tariffs and equally weighted scores), and AQoL-8D (using the Australian tariff)

Comparisons of standardised estimates					
Panel A, *n* = 1619				Panel B, *n* = 1624	
	EQ-5D-5L	EQ-5D-5L-Psychosocial	AQoL-8D	EQ-5D-5L (equally weighted score)	EQ-5D-5L- Psychosocial (equally weighted score)
Disability Severity (ref. normal)					
Mild	−0.450	−0.677	−0.720	−0.450	0.593
Moderate	−1.030	−1.364	−1.359	−1.167	−1.324
Severe	−2.128	−1.602	−1.565	−2.177	−2.001

In the regressions, the dependent variable was standardised. All models were adjusted for age and sex

## Discussion

To our knowledge, this is the first study to validate the EQ-5-D-5L-Psychosocial in a large Australian cohort with a complex and chronic disease, namely MS. A comparison of the nine-item EQ-5D-5L-Psychosocial with its two source instruments, the five-item EQ-5D-5L and the 35-item AQoL-8D, revealed that the EQ-5D-5L-Psychosocial performed well with a reduced respondent burden compared to the AQoL-8D. We also found that the EQ-5D-5L-Psychosocial and EQ-5D-5L were not interchangeable, yet the AQoL-8D and EQ-5D-5L-Psychosocial were interchangeable. These findings suggest that the EQ-5D-5L-Psychosocial is preferential to the AQoL-8D for people living with MS when taking respondent burden into account. Finally, given its larger (and negative) algorithmic range, we also found that the EQ-5D-5L is preferentially sensitive for people with severe disability, whereas the EQ-5D-5L-Psychosocial is preferentially sensitive for pwMS with no to mild disability (that is pwMS approaching full health).

### External validation of the novel EQ-5D-5L-Psychosocial

Based on our results, we conclude that the original mapping algorithm developed by Chen and Olsen in 2020 [14] is now externally validated for the first time in a large cohort of people living with MS. The new EQ-5D-5L-Psychosocial fills the psychosocial gap of the descriptive system for the EQ-5D-5L by bolting on items for vitality, sleep, relationships, and community connectedness. Previous work by our group has found that psychosocial health status is an important health outcome for people with chronic and complex disease such as people with morbid obesity who receive weight loss surgery [13], and therefore, the selection of a MAUI is crucial for eliciting relevant psychosocial health states such as sleep and social isolation.

### Using the novel EQ-5D-5L-Psychosocial for pwMS and implications for health technology assessment

Importantly, our study showed that the new items for the nine-item bolt-on instrument are essential for capturing and assessing domains of health that are relevant for pwMS. Particularly, the sleep and vitality bolt-ons were important domains of health for pwMS, as over 30% of responses were at levels 4 and 5. Failure to assess sleep quality adequately may increase the risk of not fully capturing domains of HRQoL that are important when assessing pwMS. These findings align with the literature regarding fatigue for pwMS [1]; however, sleep for pwMS is not well researched. Results generated by our group indicate that sleep is an important domain of HRQoL and that the symptomology of MS could include sleep quality as a separate symptom of MS [33, 34].

Regarding sensitivity, we found that the EQ-5D-5L-Psychosocial revealed greater discriminatory sensitivity than the AQoL-8D or EQ-5D-5L for people with no disability to mild or moderate disability. However, comparing people with severe disability, the EQ-5D-5L-Psychosocial under-performs compared to the EQ-5D-5L. There are two potential reasons for this. First, as introduced in the methods section, in this study, the indicator used for classifying disability (both PDDS and EDSS) primarily assesses mobility and physical health, which are mainly captured by the EQ-5D-5L. Second, the greater utility range of the EQ-5D-5L (i.e. −0.68 to 1.00 for EQ-5D-5L; compared to 0.046–1.00 or 0.09–1.00 for the EQ-5D-5L-Psychosocial and AQoL-8D respectively; Table 2) may increase its sensitivity. We empirically investigated this second hypothesis by using the unweighted (in essence this means equally weighted) summary score of EQ-5D-5L and EQ-5D-5L-Psychosocial (i.e. instead of using preference weight, we calculated the unweighted summary score of all dimensions for each instrument). As shown in Table 7, Panel B, the absolute magnitudes of standardised coefficients of severe disability became much closer (2.177 vs. 2.001), albeit the EQ-5D-5L still out-performed the EQ-5D-5L-Psychosocial. We also note that the stronger correlation between the EQ VAS and the AQoL-8D and EQ-5D-5L-Psychosocial than the EQ-5D-5L may be owing to the fact that more psychosocial health items are included in the classification systems of the AQoL-8D and EQ-5D-5L-Psychosocial.

Generic MAUIs are commonly used for indirect measurement of utilities, including the two source instruments for the new EQ-5D-5L-Psychosocial: the EQ-5D-5L and the AQoL-8D. Official pharmacoeconomics guidelines inform manufacturers and others about which methods to follow with respect to CUA to support applications for access, reimbursement, or pricing [12]. This is particularly important for pwMS in regard to disease-modifying therapies [35]. Although no treatment is currently available to reverse the progressive disability accumulation in MS, clinical trials of disease-modifying therapies have shown positive effects on relapse rate with some also show decreased rates of short-term disability progression in RRMS [35]. Recommendations about which instrument to use in CUA differ among countries around the world. We note that the EQ-5D-5L is recommended for CUA in over 85% of HTA guidelines [12] worldwide and that it is the most prevalent in economic evaluation. Resourcing decisions regarding disease-modifying therapies are typically based on CUA for HTAs. We suggest that the novel EQ-5D-5L-Psychosocial with its bolt-on dimensions be considered when choosing between the three MAUIs compared in this study.

## Strengths and limitations

Our study has four main strengths. First, the large and representative sample of pwMS; and when we compared those who responded to the surveys to those who did not respond, we found few material differences, with those who responded being slightly older. Second, the fact that the sample includes all levels of disability for pwMS enables the examination of floor and ceiling effects. Third, the excellent response rate for the 2020 Quality of Life Survey coupled with the generation of HSUs for 97% of participants for the three MAUIs provides confidence in the generalisability of results to the wider community of pwMS. Finally, the randomisation of questionnaires in the 2020 QoL Survey to avoid systematic responses to MAUI questions. Our study also suffered from some limitations, including the lack of comparison with a disease-specific instrument to enable concurrent validation. We note that the MS Impact Scale has been mapped to the EQ-5D-3L and SF-6Dv1 instruments [36] but there are no disease-specific instruments for a MS study population that generate a HSU. Non-respondents were slightly older than responders (by 3 years) but this difference is unlikely to be clinically meaningful. Finally, we also note that the lowest EQ-5D-5L-Psychosocial utility score is close to zero (which is similar to AQoL-8D), and therefore, no negative values are available in the value set that would translate to increased sensitivity in the more severe disability categories.

## Conclusions

Before selecting a generic MAUI, researchers should fully understand an instrument’s descriptive system. Our study found that the original mapping algorithm for the EQ-5D-5L-Psychosocial (which addresses the psychosocial gap of the descriptive system for the EQ-5D-5L) is externally validated for a large MS cohort.

The EQ-5D-5L-Psychosocial performed better than the EQ-5D-5L for the study population of pwMS with no disability to moderate disability. Additionally, when the respondent burden is taken into account, and given the interchangeability of the two instruments, the EQ-5D-5L-Psychosocial is preferential to the AQoL-8D for our study population of pwMS. This has implications regarding HTA guidelines that prescribe the EQ-5D-5L, particularly for disease-modifying therapies for pwMS. Future studies should consider further exploring the psychometric properties of other frequently used MAUIs such as the SF-6D for pwMS.

## Supplementary Information

Below is the link to the electronic supplementary material.

Supplementary file1 (DOCX 412 KB)
