# Supplementary material for: Validation of the EQ-5D-5L and psychosocial bolt-ons in a large cohort of people living with multiple sclerosis in Australia
Source: Qual Life Res. 2022 Aug 29;32(2):553–68. doi: 10.1007/s11136-022-03214-y (PMC9911481; doi:10.1007/s11136-022-03214-y)

**Supplementary materials**

**Contents**

**Supplementary Table 1:** Domains of health characteristics of the EQ-5D-5L and EQ-5D-5L-Psychosocial including bolt-on items adopted from the AQoL-8D for the EQ-5D-5L-Psychosocial.

**Supplementary Table 2:** Missing data points for the items of the EQ-5D-5L and AQoL-8D.

**Supplementary Table 3:** Comparison of participants with full health (health state utility (HSU) = 1.0) based on the EQ-5D-5L for the alternate multi-attribute utility instruments of AQoL-8D and EQ-5D-5L-Psychosocial, and the EQ-VAS.

**Supplementary Figure 1**: (A) Representation of the AQoL-8D’s structure including the instruments items, dimensions and super-dimensions (Source: aqol.com.au); (B) conceptual diagram regarding the choice of AQoL-8D bolt-on items for the EQ-5D-5LPsychosocial to fill the psychosocial gap for the EQ-5D-5L; and (B) conceptual diagram regarding the choice of AQoL-8D bolt-on items for the EQ-5D-5L-Psychosocial to fill the psychosocial gap for the EQ-5D-5L

**Supplementary Figure 2:** Distribution of individual health state utilities for EQ-5D-5L, EQ-5D-5L-Psychosocial, and AQoL-8D multi-attribute utility instruments, and the individual scores for the EQ-VAS.

**Supplementary Table 1:** Domains of health characteristics of the EQ-5D-5L and EQ-5D-5L Psychosocial including bolt-on items adopted from the AQoL-8D for the EQ-5D-5L-Psychosocial.

| **Characteristics** | **EQ-5D-5L** | **EQ-5D-5L-Psychosocial** |
| --- | --- | --- |
| Development team and year finalised | Euroqol Research Foundation (2011) | Centre for Health Economics, Monash University, Melbourne, Victoria, Australia, 2020 |
| Physical | Item 1: **Mobility**  Five levels from no problems in walking about to I am unable to walk about | Item 1: **Mobility**  Five levels from no problems in walking about to I am unable to walk about |
|  | Item 2:**Self-care**  Five levels from no problems washing or dressing myself to I am unable to wash or dress myself | Item 2:**Self-care**  Five levels from no problems washing or dressing myself to I am unable to wash or dress myself |
|  | Item 3:**Usual activities**  Five levels from no problems doing my usual activities to I am unable to do my usual activities | Item 3:**Usual activities**  Five levels from no problems doing my usual activities to I am unable to do my usual activities |
|  | Item 4: **Pain/Discomfort**  Five levels from no pain or discomfort to extreme pain or discomfort | Item 4: **Pain/Discomfort**  Five levels from no pain or discomfort to extreme pain or discomfort |
| Psychosocial | Item 5: **Anxiety/Depression**  Five levels from not anxious or depressed to extremely anxious or depressed. | Item 5: **Anxiety/Depression**  Five levels from not anxious or depressed to extremely anxious or depressed. |
| **AQoL-8D**  **Bolt-on dimensions** |  |  |
| Vitality  Bolt-on dimension from the AQoL-8D Question 1 | No | Item 6: **Vitality:** How much energy do you have to do the things you want to do?   1. I am always full of energy 2. I am usually full of energy 3. I am occasionally full of energy 4. I am usually tired and lacking energy 5. I am always tired and lacking energy |
| Social Relationships  Bolt-on dimension from the AQoL-8D Question 10 | No | Item 7: **Social relationships (family and friends):** How satisfying are your close relationships (family and friends)?   1. Very satisfying 2. Satisfying 3. Neither satisfying or dissatisfying 4. Dissatisfying 5. Unpleasant/very unpleasant |
| Sleep  Bolt-on dimension from the AQoL-8D Question 12 | No | Item 8: **Sleep:** How often do you have trouble sleeping?   1. Never 2. Almost never 3. Sometimes 4. Often 5. All the time |
| Community correctedness  Bolt-on dimension from the AQoL-8D Question 31 | No | Item 9: **Community Connectedness** How often do you feel socially isolated?   1. Never 2. Rarely 3. Sometimes 4. Often 5. Always |

**Supplementary Table 2:** Missing data points for the items of the EQ-5D-5L and AQoL-8D.

| ***EQ-5D-5L*** |  |  |
| --- | --- | --- |
| **Item** | **Missing (n=x)** | **Available**  **(n=x)** |
| *Mobility* | 8 | 1675 |
| *Self-Care* | 12 | 1671 |
| *Usual Activities* | 10 | 1673 |
| *Pain* | 12 | 1671 |
| *Anxiety and Depression* | 12 | 1671 |
| *Health State Utility* | 32 | 1651 |
| ***AQoL-8D*** |  |  |
| **Item** | **Missing** | **Available** |
| *Energy* | 8 | 1675 |
| *Socially Excluded* | 9 | 1674 |
| *Getting Around* | 11 | 1672 |
| *Role in Community* | 19 | 1664 |
| *Feel Sad* | 7 | 1676 |
| *Serious Pain* | 11 | 1672 |
| *Confidence* | 15 | 1668 |
| *Calm* | 7 | 1676 |
| *Family Relationships* | 13 | 1670 |
| *Close Relationships* | 5 | 1678 |
| *Communication* | 8 | 1675 |
| *Trouble Sleeping* | 8 | 1675 |
| *Worthless* | 6 | 1677 |
| *Angry* | 9 | 1674 |
| *Mobility* | 5 | 1678 |
| *Hurting Yourself* | 6 | 1677 |
| *Enthusiastic* | 10 | 1673 |
| *Feel Worried* | 8 | 1675 |
| *Washing* | 4 | 1679 |
| *Feel Happy* | 7 | 1676 |
| *Cope With Problems* | 6 | 1677 |
| *Pain* | 8 | 1675 |
| *Enjoy Relationships* | 8 | 1675 |
| *Pain Interfere* | 7 | 1676 |
| *Feel Pleasure* | 11 | 1672 |
| *Burden On Others* | 9 | 1674 |
| *Content* | 7 | 1676 |
| *Vision* | 10 | 1673 |
| *In Control of Life* | 3 | 1680 |
| *Help Around House* | 12 | 1671 |
| *Feel Isolated* | 6 | 1677 |
| *Hearing* | 6 | 1677 |
| *Depressed* | 4 | 1679 |
| *Close Relations Happy* | 19 | 1664 |
| *Feel Despair* | 8 | 1675 |
| *Health State Utility* | 14 | 1669 |

**Supplementary Table 3:** Comparison of participants with full health (health state utility (HSU) = 1.0) based on the EQ-5D-5L for the alternate multi-attribute utility instruments of AQoL-8D and EQ-5D-5L-Psychosocial, and the EQ-VAS.

| **Individual participant**  **(n=157)** | **EQ-5D-5L-**  **Psychosocial**  **individual HSU** | **EQ-VAS**  **(n=156)** | **AQoL-8D**  **HSU** | **EDSS** | **PDSS** | **DMT**  **Use** | **Response**  **AQ1**  **Energy**  **(n=157)** | **Response**  **AQ10**  **Relation-**  **Ships**  **(n=157)** | **Response**  **AQ12**  **Sleep**  **(n=157)** | **Response**  **AQ31**  **Commun-ity**  **(n=157)** |
| --- | --- | --- | --- | --- | --- | --- | --- | --- | --- | --- |
| 1 | 0.81 | 84 | 0.92 | 0 | 1 | Yes | 3 | 2 | 2 | 2 |
| 2 | 0.96 | 72 | 0.89 | 1 | 9 | No | 2 | 1 | 2 | 1 |
| 3 | 0.98 | 100 | 0.98 | 0 | 1 | No | 1 | 1 | 2 | 1 |
| 4 | 0.96 | 100 | 0.96 | 0 | 1 |  | 2 | 1 | 2 | 1 |
| 5 | 0.93 | 90 | 0.92 | 0 | 1 | Yes | 2 | 1 | 3 | 1 |
| 6 | 0.73 | 80 | 0.79 | 0 | 1 | Yes | 4 | 2 | 5 | 1 |
| 7 | 0.89 | 80 | 0.91 | 0 | 1 | No | 3 | 1 | 2 | 1 |
| 8 | 0.93 | 92 | 0.96 | 0 | 1 |  | 2 | 1 | 3 | 1 |
| 9 | 0.96 | 96 | 0.96 | 0 | 1 | Yes | 2 | 1 | 2 | 1 |
| 10 | 0.86 | 100 | 0.93 | 2 | 5 | No | 3 | 1 | 3 | 1 |
| 11 | 0.82 | 90 | 0.81 | 0 | 1 | Yes | 2 | 1 | 3 | 3 |
| 12 | 0.96 | 98 | 1.00 | 1 | 2 | Yes | 2 | 1 | 2 | 1 |
| 13 | 0.96 | 85 | 0.94 | 0 | 1 | Yes | 2 | 1 | 2 | 1 |
| 14 | 0.79 | 85 | 0.7 | 0 | 1 | No | 2 | 3 | 3 | 2 |
| 15 | 0.87 | 99 | 0.93 | 0 | 1 | Yes | 3 | 1 | 1 | 2 |
| 16 | 0.92 | 96 | 0.97 | 0 | 1 | Yes | 2 | 1 | 2 | 2 |
| 17 | 0.81 | 90 | 0.92 | 0 | 1 | No | 3 | 2 | 2 | 2 |
| 18 | 0.89 | 90 | 0.89 | 0 | 1 |  | 1 | 2 | 2 | 2 |
| 19 | 0.84 | 90 | 0.84 | 0 | 1 | Yes | 2 | 2 | 3 | 2 |
| 20 | 0.96 |  | 0.95 | 1 | 2 |  | 2 | 1 | 2 | 1 |
| 21 | 0.89 | 95 | 0.83 | 0 | 1 |  | 2 | 1 | 3 | 2 |
| 22 | 0.91 | 95 | 0.96 | 0 | 1 | No | 2 | 2 | 2 | 1 |
| 23 | 0.82 | 85 | 0.86 | 0 | 1 | Yes | 3 | 1 | 3 | 2 |
| 24 | 0.88 | 86 | 0.92 | 0 | 1 | No | 2 | 2 | 3 | 1 |
| 25 | 0.87 | 85 | 0.94 | 0 | 1 | No | 2 | 2 | 2 | 2 |
| 26 | 0.98 | 87 | 0.97 | 1 | 2 | Yes | 2 | 1 | 1 | 1 |
| 27 | 0.89 | 80 | 0.88 | 0 | 1 |  | 2 | 1 | 3 | 2 |
| 28 | 0.96 | 98 | 0.96 | 0 | 1 | No | 2 | 1 | 2 | 1 |
| 29 | 0.92 | 99 | 0.95 | 0 | 1 | Yes | 2 | 1 | 2 | 2 |
| 30 | 1.00 | 100 | 1.00 | 0 | 1 | No | 1 | 1 | 1 | 1 |
| 31 | 0.91 | 90 | 0.94 | 0 | 1 | Yes | 2 | 1 | 4 | 1 |
| 32 | 0.80 | 92 | 0.84 | 0 | 1 | Yes | 3 | 1 | 4 | 2 |
| 33 | 0.91 | 95 | 0.94 | 1 | 2 | No | 2 | 2 | 2 | 1 |
| 34 | 0.96 | 85 | 0.98 | 0 | 1 |  | 2 | 1 | 2 | 1 |
| 35 | 0.93 | 94 | 0.96 | 0 | 1 | Yes | 2 | 1 | 3 | 1 |
| 36 | 0.76 | 90 | 0.69 | 0 | 1 | No | 3 | 2 | 4 | 2 |
| 37 | 0.91 | 95 | 0.98 | 0 | 1 | Yes | 2 | 1 | 4 | 1 |
| 38 | 0.82 | 80 | 0.85 | 1 | 2 | Yes | 2 | 1 | 3 | 3 |
| 39 | 0.95 | 93 | 0.98 | 0 | 1 | Yes | 1 | 1 | 3 | 1 |
| 40 | 0.96 | 85 | 0.98 | 0 | 1 | Yes | 2 | 1 | 2 | 1 |
| 41 | 0.81 | 90 | 0.84 | 0 | 1 | Yes | 2 | 3 | 2 | 2 |
| 42 | 0.96 | 95 | 0.99 | 0 | 1 | Yes | 2 | 1 | 2 | 1 |
| 43 | 0.91 | 78 | 0.95 | 1 | 2 | No | 2 | 1 | 4 | 1 |
| 44 | 0.79 | 76 | 0.79 | 1 | 9 | Yes | 4 | 1 | 2 | 2 |
| 45 | 0.96 | 90 | 0.96 |  |  | No | 2 | 1 | 2 | 1 |
| 46 | 0.84 | 91 | 0.95 | 0 | 1 | Yes | 2 | 2 | 3 | 2 |
| 47 | 0.92 | 84 | 0.87 | 0 | 1 |  | 2 | 1 | 2 | 2 |
| 48 | 0.80 | 92 | 0.88 | 0 | 1 | Yes | 2 | 2 | 2 | 3 |
| 49 | 0.77 | 80 | 0.81 | 1 | 9 | No | 3 | 3 | 3 | 1 |
| 50 | 0.88 | 95 | 0.92 | 0 | 1 | Yes | 2 | 2 | 3 | 1 |
| 51 | 0.92 | 90 | 0.95 | 1 | 9 | No | 2 | 1 | 2 | 2 |
| 52 | 0.84 | 100 | 0.97 | 0 | 1 | Yes | 3 | 1 | 4 | 1 |
| 53 | 0.96 | 90 | 0.99 | 0 | 1 | Yes | 2 | 1 | 2 | 1 |
| 54 | 0.91 | 92 | 0.90 | 0 | 1 |  | 2 | 2 | 2 | 1 |
| 55 | 0.96 | 81 | 0.97 | 0 | 1 | Yes | 2 | 1 | 2 | 1 |
| 56 | 0.89 | 88 | 0.86 | 1 | 2 |  | 3 | 1 | 2 | 1 |
| 57 | 0.96 | 91 | 0.9 | 1 | 2 | No | 2 | 1 | 2 | 1 |
| 58 | 0.96 | 96 | 0.95 | 0 | 1 | No | 2 | 1 | 2 | 1 |
| 59 | 0.88 | 95 | 0.92 | 0 | 1 | Yes | 2 | 2 | 3 | 1 |
| 60 | 0.93 | 90 | 0.95 | 0 | 1 | No | 2 | 1 | 3 | 1 |
| 61 | 0.98 | 90 | 0.98 | 0 | 1 | No | 2 | 1 | 1 | 1 |
| 62 | 0.96 | 100 | 0.98 | 0 | 1 | Yes | 2 | 1 | 2 | 1 |
| 63 | 0.93 | 75 | 0.91 | 0 | 1 | Yes | 2 | 1 | 3 | 1 |
| 64 | 0.73 | 97 | 0.83 | 0 | 1 | Yes | 3 | 1 | 5 | 3 |
| 65 | 0.92 | 90 | 0.89 | 2 | 5 | Yes | 2 | 1 | 2 | 2 |
| 66 | 0.88 | 100 | 0.97 | 1 | 2 | No | 2 | 2 | 3 | 1 |
| 67 | 0.91 | 95 | 0.93 | 0 | 1 | No | 2 | 2 | 2 | 1 |
| 68 | 0.98 | 88 | 0.97 | 0 | 1 |  | 1 | 1 | 2 | 1 |
| 69 | 0.92 | 16 | 0.91 | 0 | 1 | Yes | 2 | 1 | 2 | 2 |
| 70 | 0.92 | 98 | 0.96 | 0 | 1 | Yes | 2 | 1 | 2 | 2 |
| 71 | 0.74 | 85 | 0.73 | 1 | 2 | Yes | 3 | 2 | 2 | 3 |
| 72 | 0.98 | 75 | 0.99 | 0 | 1 |  | 2 | 1 | 1 | 1 |
| 73 | 0.96 | 75 | 0.94 | 0 | 1 | No | 2 | 1 | 2 | 1 |
| 74 | 0.94 | 90 | 0.98 | 0 | 1 | No | 2 | 1 | 1 | 2 |
| 75 | 0.93 | 90 | 0.94 | 0 | 1 |  | 2 | 2 | 1 | 1 |
| 76 | 0.96 | 88 | 0.92 | 0 | 1 | Yes | 2 | 1 | 2 | 1 |
| 77 | 0.96 | 98 | 0.98 | 0 | 1 | Yes | 2 | 1 | 2 | 1 |
| 78 | 0.91 | 100 | 0.88 | 0 | 1 | Yes | 2 | 2 | 2 | 1 |
| 79 | 0.82 | 85 | 0.79 | 0 | 1 | Yes | 2 | 2 | 5 | 2 |
| 80 | 0.87 | 95 | 0.86 | 0 | 1 | Yes | 2 | 2 | 2 | 2 |
| 81 | 0.96 | 91 | 0.95 | 0 | 1 |  | 2 | 1 | 2 | 1 |
| 82 | 0.87 | 98 | 0.95 | 0 | 1 | Yes | 2 | 2 | 2 | 2 |
| 83 | 0.93 | 95 | 0.96 | 0 | 1 | Yes | 2 | 1 | 3 | 1 |
| 84 | 0.98 | 100 | 1.00 | 1 | 9 |  | 1 | 1 | 2 | 1 |
| 85 | 0.94 | 100 | 0.94 | 0 | 1 | Yes | 2 | 1 | 1 | 2 |
| 86 | 0.93 | 90 | 0.94 | 0 | 1 | Yes | 1 | 2 | 2 | 1 |
| 87 | 0.89 | 100 | 0.93 | 0 | 1 | Yes | 2 | 1 | 3 | 2 |
| 88 | 0.96 | 80 | 0.99 | 0 | 1 | Yes | 2 | 1 | 2 | 1 |
| 89 | 0.93 | 92 | 0.96 | 0 | 1 | Yes | 2 | 1 | 3 | 1 |
| 90 | 0.89 | 100 | 0.92 | 0 | 1 | No | 3 | 1 | 2 | 1 |
| 91 | 1.00 | 80 | 1.00 | 0 | 1 | Yes | 1 | 1 | 1 | 1 |
| 92 | 0.93 | 70 | 0.99 | 0 | 1 |  | 2 | 2 | 1 | 1 |
| 93 | 0.96 | 95 | 0.97 | 0 | 1 |  | 2 | 1 | 2 | 1 |
| 94 | 0.84 | 98 | 0.88 | 1 | 2 | Yes | 2 | 2 | 3 | 2 |
| 95 | 0.85 | 90 | 0.88 | 0 | 1 | Yes | 2 | 1 | 2 | 3 |
| 96 | 0.91 | 85 | 0.93 | 0 | 1 | Yes | 2 | 1 | 4 | 1 |
| 97 | 0.955 | 80 | 0.98 | 0 | 1 | Yes | 2 | 1 | 2 | 1 |
| 98 | 0.86 | 98 | 0.82 | 1 | 2 | Yes | 3 | 1 | 3 | 1 |
| 99 | 0.78 | 80 | 0.66 | 1 | 9 | Yes | 3 | 2 | 3 | 2 |
| 100 | 0.81 | 85 | 0.78 | 0 | 1 | Yes | 3 | 2 | 2 | 2 |
| 101 | 0.80 | 90 | 0.76 | 1 | 2 | Yes | 3 | 3 | 2 | 1 |
| 102 | 0.93 | 95 | 0.97 | 0 | 1 | Yes | 2 | 1 | 3 | 1 |
| 103 | 0.87 | 72 | 0.78 | 1 | 2 | Yes | 3 | 2 | 1 | 1 |
| 104 | 0.87 | 95 | 0.92 | 0 | 1 |  | 2 | 2 | 2 | 2 |
| 105 | 0.91 | 85 | 0.88 | 0 | 1 | Yes | 2 | 1 | 4 | 1 |
| 106 | 0.98 | 96 | 1.00 | 0 | 1 | Yes | 2 | 1 | 1 | 1 |
| 107 | 0.98 | 97 | 0.99 | 0 | 1 | Yes | 1 | 1 | 2 | 1 |
| 108 | 0.98 | 85 | 0.97 | 1 | 2 | No | 2 | 1 | 1 | 1 |
| 109 | 1.00 | 98 | 1.00 | 0 | 1 | No | 1 | 1 | 1 | 1 |
| 110 | 0.94 | 100 | 0.99 | 0 | 1 | Yes | 2 | 1 | 1 | 2 |
| 111 | 0.95 | 100 | 1.00 | 0 | 1 | Yes | 1 | 1 | 3 | 1 |
| 112 | 0.92 | 93 | 0.95 | 0 | 1 |  | 2 | 1 | 2 | 2 |
| 113 | 0.88 | 100 | 0.88 | 0 | 1 | No | 2 | 2 | 3 | 1 |
| 114 | 1.00 | 100 | 1.00 | 0 | 1 |  | 1 | 1 | 1 | 1 |
| 115 | 0.87 | 84 | 0.87 | 2 | 3 | Yes | 2 | 2 | 2 | 2 |
| 116 | 0.93 | 90 | 0.94 | 0 | 1 | No | 2 | 1 | 3 | 1 |
| 117 | 0.98 | 96 | 1.00 | 0 | 1 |  | 1 | 1 | 2 | 1 |
| 118 | 0.96 | 86 | 0.96 | 0 | 1 | Yes | 2 | 1 | 2 | 1 |
| 119 | 0.78 | 70 | 0.66 | 2 | 3 | Yes | 3 | 2 | 3 | 2 |
| 120 | 0.78 | 90 | 0.86 | 0 | 1 | Yes | 3 | 2 | 3 | 2 |
| 121 | 0.86 | 94 | 0.95 | 0 | 1 | No | 1 | 2 | 3 | 2 |
| 122 | 0.94 | 80 | 0.93 | 0 | 1 | Yes | 2 | 1 | 2 | 2 |
| 123 | 0.94 | 90 | 0.9 | 0 | 1 | Yes | 2 | 1 | 2 | 2 |
| 124 | 0.87 | 99 | 0.67 | 0 | 1 |  | 2 | 1 | 4 | 2 |
| 125 | 0.98 | 74 | 0.97 | 0 | 1 | Yes | 2 | 1 | 1 | 1 |
| 126 | 0.85 | 10 | 0.89 | 0 | 1 | No | 3 | 1 | 2 | 2 |
| 127 | 0.96 | 90 | 1.00 | 1 | 2 |  | 2 | 1 | 2 | 1 |
| 128 | 0.93 | 95 | 0.92 | 0 | 1 |  | 2 | 1 | 3 | 1 |
| 129 | 1.00 | 100 | 1.00 | 0 | 1 | Yes | 1 | 1 | 1 | 1 |
| 130 | 1.00 | 97 | 1.00 | 0 | 1 | Yes | 1 | 1 | 1 | 1 |
| 131 | 0.91 | 95 | 0.79 | 0 | 1 |  | 3 | 1 | 1 | 1 |
| 132 | 0.64 | 85 | 0.65 | 1 | 2 |  | 3 | 3 | 4 | 3 |
| 133 | 0.98 | 98 | 0.97 | 0 | 1 | Yes | 1 | 1 | 2 | 1 |
| 134 | 0.93 | 98 | 0.92 | 1 | 2 | Yes | 2 | 1 | 3 | 1 |
| 135 | 0.96 | 95 | 0.97 | 0 | 1 | No | 2 | 1 | 2 | 1 |
| 136 | 0.96 | 0 | 0.95 | 0 | 1 | No | 2 | 1 | 2 | 1 |
| 137 | 0.96 | 85 | 0.94 | 0 | 1 | Yes | 2 | 1 | 2 | 1 |
| 138 | 0.62 | 60 | 0.65 | 2 | 3 |  | 3 | 4 | 3 | 4 |
| 139 | 0.93 | 15 | 0.93 | 0 | 1 | Yes | 2 | 1 | 3 | 1 |
| 140 | 0.88 | 82 | 0.9 | 0 | 1 | Yes | 2 | 2 | 3 | 1 |
| 141 | 0.93 | 85 | 0.94 | 0 | 1 |  | 2 | 1 | 3 | 1 |
| 142 | 0.65 | 80 | 0.71 | 0 | 1 | Yes | 4 | 2 | 3 | 3 |
| 143 | 1.00 | 90 | 1.00 | 0 | 1 | Yes | 1 | 1 | 1 | 1 |
| 144 | 0.91 | 95 | 0.97 | 0 | 1 | Yes | 2 | 2 | 2 | 1 |
| 145 | 0.81 | 95 | 0.69 | 1 | 2 | Yes | 2 | 4 | 2 | 2 |
| 146 | 0.72 | 25 | 0.63 | 1 | 9 | Yes | 4 | 2 | 3 | 2 |
| 147 | 0.76 | 90 | 0.83 | 1 | 2 | Yes | 4 | 2 | 3 | 1 |
| 148 | 0.84 | 75 | 0.85 | 1 | 2 | Yes | 2 | 2 | 3 | 2 |
| 149 | 0.93 | 99 | 0.89 | 0 | 1 | No | 2 | 1 | 3 | 1 |
| 150 | 0.78 | 90 | 0.88 | 0 | 1 | No | 3 | 2 | 3 | 2 |
| 151 | 0.78 | 84 | 0.87 | 1 | 2 | No | 3 | 2 | 3 | 2 |
| 152 | 0.84 | 13 | 0.93 | 0 | 1 | Yes | 3 | 1 | 4 | 1 |
| 153 | 0.85 | 90 | 0.87 | 1 | 2 |  | 2 | 1 | 2 | 3 |
| 154 | 0.98 | 90 | 1 | 0 | 1 | Yes | 2 | 1 | 1 | 1 |
| 155 | 0.91 | 88 | 0.91 | 2 | 3 | 0 | 2 | 2 | 2 | 1 |
| 156 | 0.91 | 84 | 0.87 | 1 | 2 | Yes | 2 | 2 | 2 | 1 |
| 157 | 0.95 | 100 | 1.00 | 0 | 1 | Yes | 1 | 1 | 3 | 1 |
| Notes: DMT, disease modifying therapy; EDSS Expanded Disability Status Scale our classifications of MS-related disability severity classified as: no disability (EDSS level: 0), mild disability (EDSS 1–3.5), moderate disability (EDSS 4–6) and severe disability (EDSS 6.5–9.5); HSU, health state utility; PDDS Patient Determined Disease Steps (scale 1-9). | | | | | | | | | | |

**Supplementary Figure 1**: (A) Representation of the AQoL-8D’s structure including the instruments items, dimensions and super-dimensions (Source: aqol.com.au); (B) conceptual diagram regarding the choice of AQoL-8D bolt-on items for the EQ-5D-5LPsychosocial to fill the psychosocial gap for the EQ-5D-5L source *Chen and Olsen* 2020.

**A**


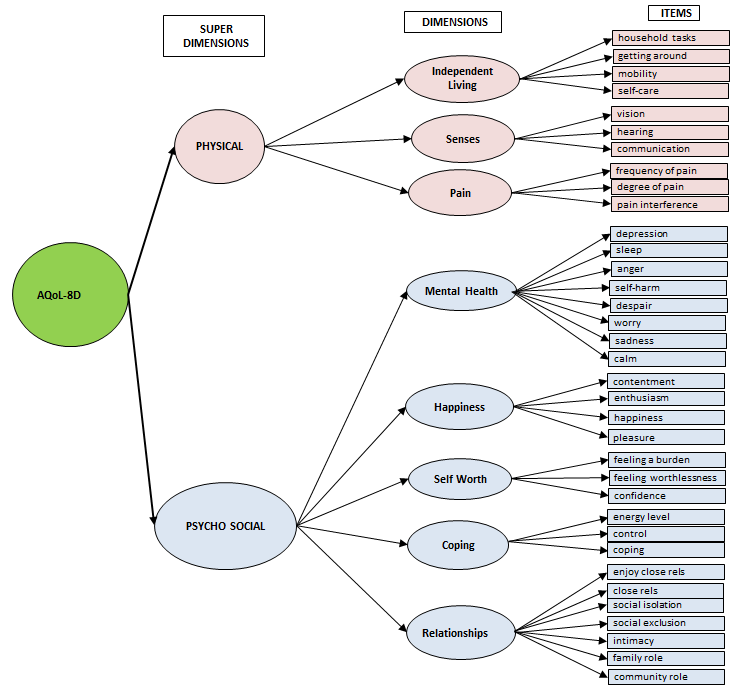


Source: AQoL http//aqol.com.au

**Supplementary Figure 1:** (B) conceptual diagram regarding the choice of AQoL-8D bolt-on items for the EQ-5D-5L-Psychosocial to fill the psychosocial gap for the EQ-5D-5L

**(B)**


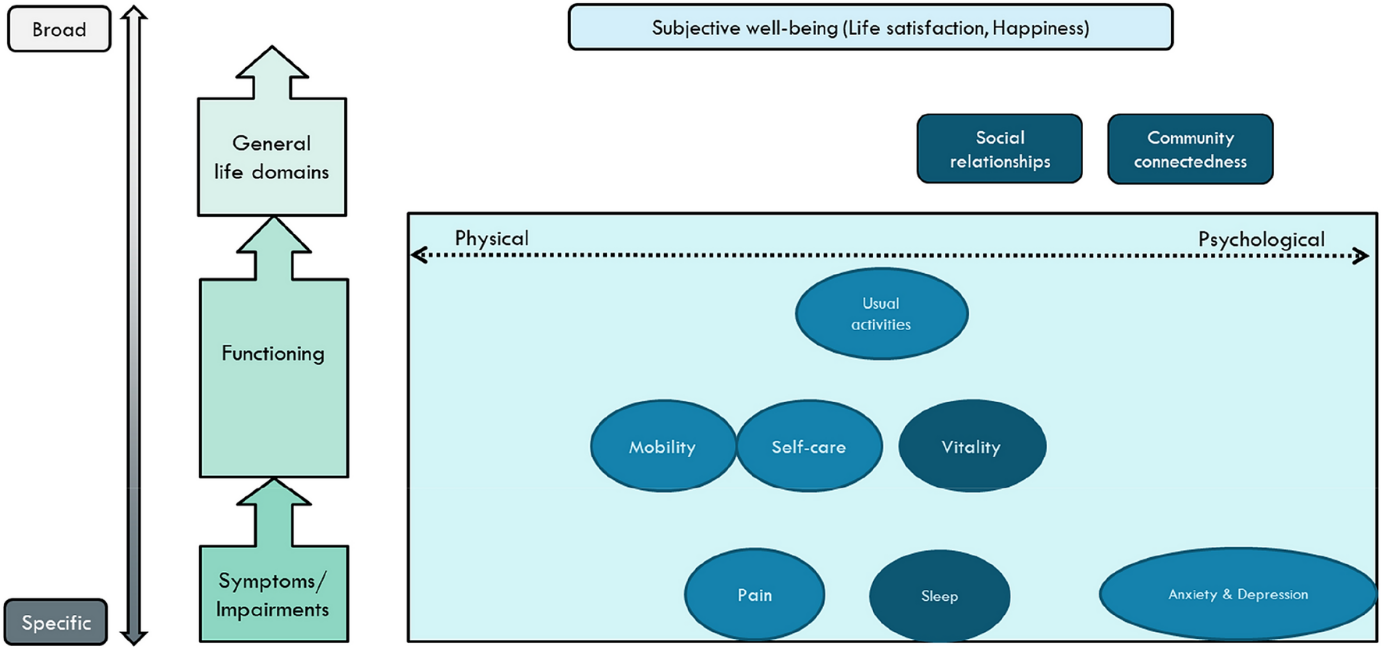


*Source: Chen and Olsen 2020, Quality of Life Research*

**Supplementary Figure 2:** Distribution of individual health state utilities for EQ-5D-5L, EQ-5D-5L-Psychosocial, and AQoL-8D multi-attribute utility instruments, and the individual scores for the EQ-VAS.


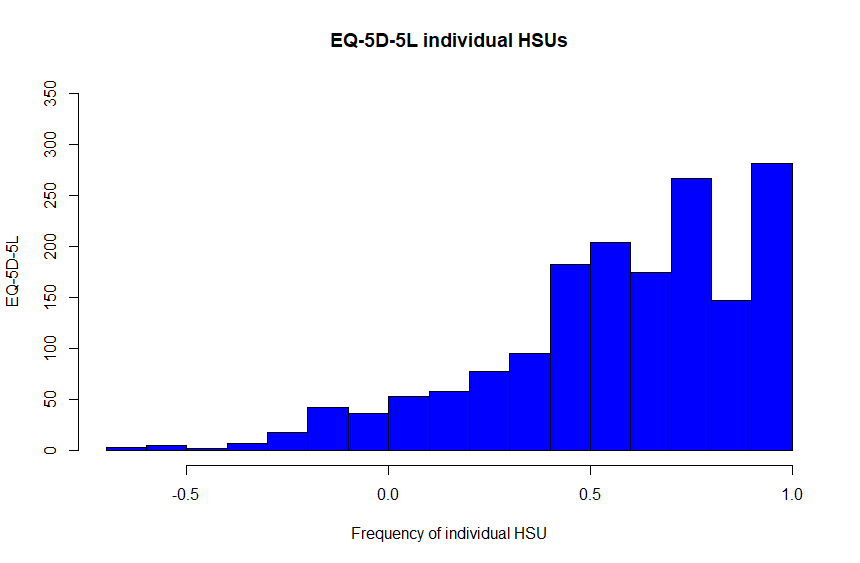


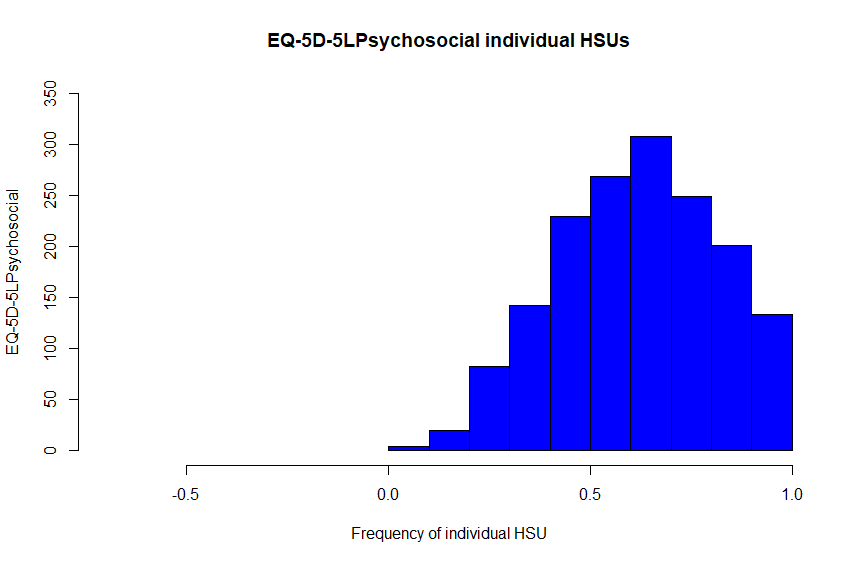


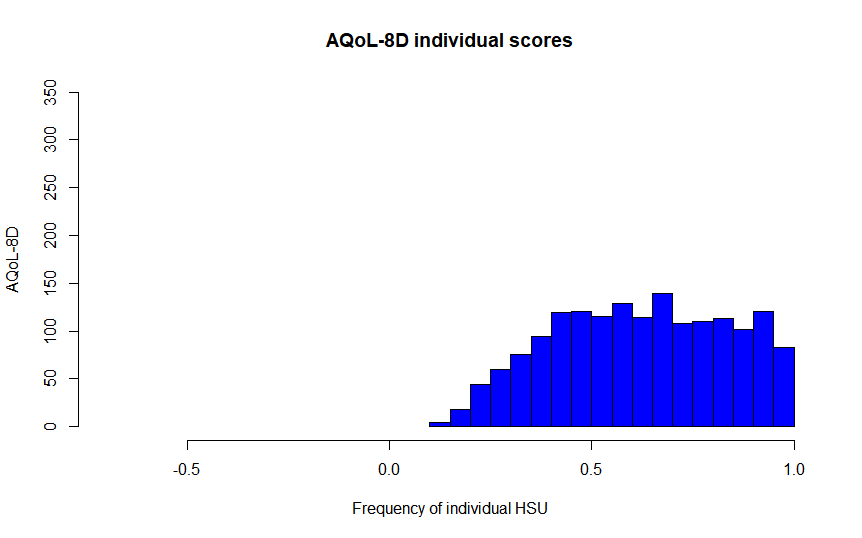


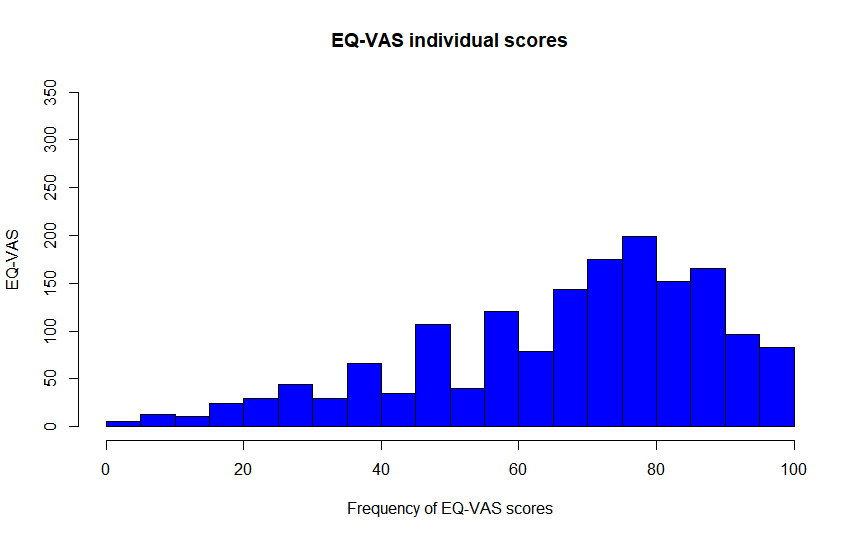

Supplement: Supplementary file 1 — Supplementary file1 (DOCX 412 KB) [file 11136_2022_3214_MOESM1_ESM.docx]
